# The effects of a 2-year physical activity and dietary intervention on plasma lipid concentrations in children: the PANIC Study

**DOI:** 10.1007/s00394-020-02260-x

**Published:** 2020-05-04

**Authors:** Aino-Maija Eloranta, Taisa Sallinen, Anna Viitasalo, Niina Lintu, Juuso Väistö, Henna Jalkanen, Tuomo T. Tompuri, Sonja Soininen, Eero A. Haapala, Sanna Kiiskinen, Theresia M. Schnurr, Tuomas O. Kilpeläinen, Santtu Mikkonen, Kai Savonen, Mustafa Atalay, Soren Brage, David E. Laaksonen, Virpi Lindi, Jyrki Ågren, Ursula Schwab, Jarmo Jääskeläinen, Timo A. Lakka

**Affiliations:** 1 Institute of Public Health and Clinical Nutrition, School of Medicine, University of Eastern Finland, P.O. Box 1627, 70211 Kuopio, Finland; 2 Institute of Biomedicine, School of Medicine, University of Eastern Finland, Kuopio, Finland; 3 Department of Clinical Physiology and Nuclear Medicine, School of Medicine, Kuopio University Hospital, University of Eastern Finland, Kuopio, Finland; 4 Social and Health Center, City of Varkaus, Varkaus, Finland; 5 Faculty of Sport and Health Sciences, University of Jyväskylä, Jyväskylä, Finland; 6 Novo Nordisk Foundation Center for Basic Metabolic Research, University of Copenhagen, Copenhagen, Denmark; 7 Department of Applied Physics, University of Eastern Finland, Kuopio, Finland; 8 Kuopio Research Institute of Exercise Medicine, Kuopio, Finland; 9 MRC Epidemiology Unit, University of Cambridge, Cambridge, UK; 10 Department of Medicine, Endocrinology and Clinical Nutrition, Kuopio University Hospital, Kuopio, Finland; 11 University of Eastern Finland Library Kuopio, Kuopio, Finland; 12 Department of Pediatrics, School of Medicine, Kuopio University Hospital and University of Eastern Finland, Kuopio, Finland

**Keywords:** Intervention, Children, Lipoproteins, LDL cholesterol, Physical activity, Diet

## Abstract

**Purpose:**

We studied the effects of a physical activity and dietary intervention on plasma lipids in a general population of children. We also investigated how lifestyle changes contributed to the intervention effects.

**Methods:**

We carried out a 2-year controlled, non-randomized lifestyle intervention study among 504 mainly prepubertal children aged 6–9 years at baseline. We assigned 306 children to the intervention group and 198 children to the control group. We assessed plasma concentrations of total, LDL, HDL, and VLDL cholesterol, triglycerides, HDL triglycerides, and VLDL triglycerides. We evaluated the consumption of foods using 4-day food records and physical activity using a movement and heart rate sensor. We analyzed data using linear mixed-effect models adjusted for age at baseline, sex, and pubertal stage at both time points. Furthermore, specific lifestyle variables were entered in these models.

**Results:**

Plasma LDL cholesterol decreased in the intervention group but did not change in the control group ( − 0.05 vs. 0.00 mmol/L, regression coefficient (β) =  − 0.0385, *p* = 0.040 for group*time interaction). This effect was mainly explained by the changes in the consumption of high-fat vegetable oil-based spreads (β =  − 0.0203, + 47% change in β) and butter-based spreads (β =  − 0.0294, + 30% change in β), moderate-to-vigorous physical activity (β =  − 0.0268, + 30% change in β), light physical activity (β =  − 0.0274, + 29% change in β) and sedentary time (β =  − 0.0270, + 30% change in β). The intervention had no effect on other plasma lipids.

**Conclusion:**

Lifestyle intervention resulted a small decrease in plasma LDL cholesterol concentration in children. The effect was explained by changes in quality and quantity of dietary fat and physical activity.

**Clinical Trial Registry Number:**

NCT01803776, ClinicalTrials.gov

## Introduction

Pathophysiological processes underlying the development of cardiovascular diseases are known to begin already in childhood [1]. Higher plasma total and LDL cholesterol concentrations in childhood have been reported to be associated with increased carotid artery intima–media thickness, an indicator of preclinical atherosclerosis, in adulthood [2, 3]. The development of cardiovascular diseases could potentially be prevented by sufficient quantity and intensity of physical activity and a healthy diet in early childhood onwards [4].

A meta-analysis observed that physical activity interventions decreased plasma triglycerides by 12% [5]. Moreover, a dietary intervention study showed a decrease in plasma LDL cholesterol both at 1-year timepoint ( − 4.8 mg/dL) and 3-year timepoint ( − 3.3 mg/dL) in children with hypercholesterolemia [6]. However, other lifestyle intervention studies have shown little or no effects on plasma concentrations of lipids in children [5, 7, 8]. One reason for varying effects of lifestyle interventions on plasma lipids and lipoproteins may be the differences in the contents of the interventions. Therefore, it is important to study which lifestyle changes explain the intervention effects on plasma concentrations of lipids. Moreover, there are few studies on the effects of physical activity interventions [5] and dietary interventions [9], and no studies on the effects of combined physical activity and dietary interventions on plasma lipid concentrations in general populations of children.

We carried out a 2-year lifestyle intervention study to investigate the effects of the combined physical activity and dietary intervention on plasma concentrations of total cholesterol, LDL cholesterol, HDL cholesterol, VLDL cholesterol, triglycerides, HDL triglycerides, and VLDL triglycerides in a population sample of mainly prepubertal children. We also studied how changes in physical activity, sedentary behavior, and diet contributed to the possible effects of the intervention on plasma concentrations of lipids. This information could be used in planning effective and targeted lifestyle interventions for the prevention of cardiovascular diseases since childhood.

## Methods

### Study design and participants

The Physical Activity and Nutrition in Children (PANIC) Study is a controlled lifestyle intervention study investigating the effects of a combined physical activity and dietary intervention on cardiometabolic risk factors in a population sample of children from the city of Kuopio, Finland. The Research Ethics Committee of the Hospital District of Northern Savo approved the study protocol in 2006 (Statement 69/2006). A written informed consent was acquired from the parent or caregiver of each child and every child provided assent to participation. This consent or assent could be revoked by the parent or child at any time.

**Fig. 1:**
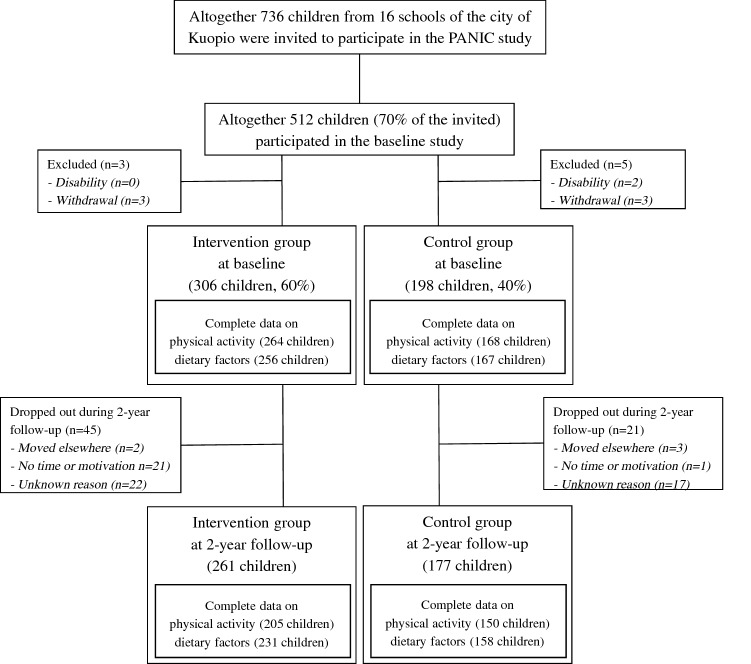
Flow chart of the Physical Activity and Nutrition in Children (PANIC) Study

We invited 736 children 6–9 years of age who started the first grade in 16 primary schools of Kuopio in 2007–2009 (Fig. 1). Altogether 512 children (248 girls, 264 boys), who accounted for 70% of those invited, participated in the baseline examinations in 2007–2009. The participants did not differ in age, sex, or body mass index standard deviation score (BMI-SDS) from all children who started the first grade in the city of Kuopio in 2007–2009 based on data from the standard school health examinations performed for all Finnish children before the first grade. We excluded six children from the study at baseline because of physical disabilities that could hamper participation in the intervention or no time or motivation to attend in the study. We also excluded two children whose parents later withdrew their permission to use the data of their children.

We cluster divided the remaining 504 children into an intervention group (306 children) and a control group (198 children) according to schools to be able to organize after-school exercise clubs carried out at school premises only for the intervention group and to avoid non-intentional intervention in the control group. We also matched the intervention and control group according to the size (large vs. small) and location (urban vs. rural) of the schools to minimize sociodemographic differences in baseline characteristics between the groups. We included more children in the intervention group than in the control group because of a larger number of dropouts expected in the intervention group and to retain a sufficient statistical power for the comparison between the groups.

All the measurements conducted at baseline were repeated at 2-year follow-up. Among 504 children who participated in the baseline examinations, 438 (87%) children also attended the 2-year follow-up examinations, 261 children from the intervention group (85%) and 177 children from the control group (89%). The median (interquartile range) follow-up time was 2.11 (interquartile range 2.07–2.16) years in intervention group and 2.13 (interquartile range 2.05–2.22) years in control group. Data on physical activity were available for 432 children (216 girls, 216 boys) at baseline and for 355 children (181 girls, 174 boys) at 2-year follow-up. Data on diet were available for 423 children (206 girls, 217 boys) at baseline and for 389 children (185 girls, 204 boys) at 2-year follow-up (Fig. 1).

### Physical activity and dietary intervention

The goals of this 2-year individualized and family-based physical activity and dietary intervention were based on national recommendations for physical activity and nutrition [10–12]. The goals were to (1) increase total physical activity by emphasizing its diversity, (2) decrease total and particularly screen-based sedentary behavior, (3) decrease the consumption of significant sources of saturated fat and particularly high-fat dairy and meat products, (4) increase the consumption of significant sources of unsaturated fat and particularly high-fat vegetable oil-based margarines, vegetable oils, and fish, (5) increase the consumption of vegetables, fruits, and berries, (6) increase the consumption of significant sources of fiber and particularly whole grain products, (7) decrease the consumption of significant sources of sugar and particularly sugar-sweetened beverages, sugar-sweetened dairy products, and candy, (8) decrease the consumption of significant sources of salt and the use of salt in cooking, and 9) avoid excessive energy intake, for example, by recommending regular consumption of main meals and avoiding frequent snacking.

The intervention included 6 physical activity and diet counseling sessions consisting of 30–45 min of physical activity counseling and 30–45 min of dietary counseling for the children and their parents during the 2-year follow-up [13]. The 6 counseling sessions occurred 0.5, 1.5, 3, 6, 12, and 18 months after baseline. In these counseling sessions, the children and their parents received individualized advice from a specialist in exercise medicine and a clinical nutritionist on how to increase physical activity, decrease sedentary behavior, and improve diet among children in everyday conditions. Each counseling session had a specific topic on physical activity, sedentary behavior, and diet according to the goals of the intervention and included practical tasks on these topics for the children. In the counseling sessions, the children and their parents also received fact sheets on physical activity, sedentary behavior, and diet, verbal and written information on opportunities to exercise in the city of Kuopio, and some material support for physical activity, such as exercise equipments and allowance for playing indoor sports. Of all 306 children in the intervention group, 266 (87%) participated in all 6 counseling sessions, 281 (92%) in at least 5 counseling sessions, and 295 (96%) in at least 4 counseling sessions.

We encouraged the children in the intervention group, particularly those who did not attend organized sports or exercise, to participate in after-school exercise clubs supervised by trained exercise instructors. Of all 306 children in the intervention group, 254 (87%) children participated in at least one of the after-school exercise clubs and 124 (41%) children attended the after-school exercise clubs at least once a month.

The children and their parents in the control group received general verbal and written advice on health improving physical activity and diet but no active intervention.

### Assessment of body height, body weight, body fat percentage and puberty

Body height and weight were assessed after the children fasted for 12 h. Body height was assessed using a wall-mounted stadiometer and body weight using the InBody® 720 bioelectrical impedance device (Biospace, Seoul, Korea), with the weight assessment integrated into the system. We computed age- and sex-standardized BMI-SDS using Finnish references [14] and defined overweight and obesity using the International Obesity Task Force criteria for children that are based on centile curves passing through adult BMI cutoffs at 25 for overweight and at 30 for obesity [15]. We measured body fat percentage with the children being in the supine position, having emptied the bladder, and being in light clothing by dual-energy X-ray absorptiometry using the Lunar Prodigy Advance® dual-energy X-ray absorptiometry device (GE Medical Systems, Madison, WI). A research physician classified the girls as entered clinical puberty if their breast development had started and the boys if their testicular volume assessed by an orchidometer was ≥ 4 ml [16] according to the criteria described by Tanner [17].

### Assessment of lipids and lipoproteins

Fasting plasma concentrations of total cholesterol, LDL cholesterol, HDL cholesterol, and triglycerides were analyzed using a clinical chemistry analyzer (Hitachi High Technology Co, Tokyo, Japan). Before analysis, VLDL was separated by ultracentrifugation (37 000 rpm, 15 h). Concentrations of plasma total cholesterol and triglycerides as well as LDL, HDL, and VLDL cholesterol and triglycerides were measured by colorimetric enzymatic assays.

### Assessment of diet

We assessed the consumption of food and drinks using one food record at baseline and one food record at 2-year follow-up [18]. The food record at both timepoints covered 4 predefined and consecutive days, including at least 1 weekend day. In addition at 2-year follow-up, 0.5% of all food records covered 3 days and consisted of 2 weekdays and 1 weekend day and they were also included in the analyses. Clinical nutritionists checked the filled food records together with the family and added any missing information. We calculated food consumption and nutrient intake using the Micro Nutrica® dietary analysis software, Version 2.5. The software is based on detailed information about the nutrient content of foods in Finland and other countries [19]. Moreover, a clinical nutritionist updated the software by adding new food items and products with their precise nutrient content based on new data in the Finnish food composition database [20] or received from the producers.

### Assessment of physical activity and sedentary behavior

Physical activity and sedentary behavior were assessed by a combined movement and heart rate sensor (Actiheart®; CamNtech, Cambridge, UK) [21]. The sensor was attached on the chest with 2 standard electrocardiogram electrodes and set to record in 60-s epochs. The participants were requested to wear the sensor continuously for a minimum of 4 consecutive days, including 2 weekdays and 2 weekend days, and instructed to carry on with their usual behavior and to wear the sensor during all daily activities, including sleep, shower, sauna, and swimming. Upon retrieving the sensor, heart rate data were first cleaned [22], then individually calibrated with parameters from a previously performed maximal cycle ergometer exercise test [23] and combined with trunk acceleration using branched equation modelling to produce intensity time-series [24, 25]. Whilst minimizing diurnal bias caused by any potential non-wear episodes [26], physical activity energy expenditure was calculated by time-integration of the intensity time-series, and the time distribution of activity intensity was generated using standard metabolic equivalent of tasks (METs) in 0.5 increments. For these analyses, the equivalent of 3.5 mL O_2_ /kg/min (71.2 J/kg/min) was used to define 1 MET, and data were summarized as sedentary behavior (≤ 1.5 METs excluding sleep), light intensity physical activity (> 1.5 and ≤ 4.0 METs), and moderate-to-vigorous physical activity (> 4 METs). Physical activity records were included in the analysis if there was a minimum of 48 h of activity recording in weekday and weekend day hours that included at least 12 h from morning (3 am–9 am), noon (9 am–3 pm), afternoon (3 pm–9 pm), and night (9 pm–3 am) to avoid potential bias from over-representing specific times and activities of the days.

### Assessment of parental education

We collected data on parental education level by a questionnaire. Parental education level was defined as the highest completed or ongoing degree of the parents (vocational school or less; polytechnic or university).

### Power calculations

We determined the number of children required to detect at least a 0.30 standard deviation difference in the primary outcomes between the intervention group (60% of children) and the control group (40% of children) with a power of 80% and a two-sided p-value for the difference between the groups of 0.05, allowing for a 20% loss to follow-up or missing data. According to these power calculations, we would need a sample size of at least 275 children in the intervention group and at least 183 children in the control group at baseline.

### Statistical methods

We performed all statistical analyses using the IBM SPSS Statistics® software, Version 25.0 (IBM Corp., Armonk, NY, USA). *P*-values < 0.05 were used to indicate statistical significance, based on two-sided testing. The distributions of each continuous variable were examined by observing histograms and logarithmic transformation was performed for plasma VLDL cholesterol, plasma triglyceride, and plasma VLDL triglycerides, because of their skewed distribution. We compared baseline characteristics between the groups by the *t*-test for independent samples or the Chi-Square test. We studied the effects of the intervention on plasma lipids and lipoproteins using the intention-to-treat principle including all 504 children in the statistical analyses. We used the linear mixed-effects model analyses according to a three-level structure, i.e., repeated outcome measures (baseline and follow-up) were clustered within children who were considered as subjects in the mixed model structure and children were clustered within schools. The linear mixed-effect models are especially suitable for analyzing longitudinal datasets containing correlated and unbalanced data. We started with a model adjusted for age at baseline, sex, and pubertal stage at both time points, including main effects for time and time-by-study group interaction: OUTCOME_it_ = (*β*_0_ + u_i_) + *β*_1_age + *β*_2_sex + *β*_3_ cpubertal stage + (*β*_4_ + *v*_i_)time + *β*_5_study group x time + *ε*_it_, where OUTCOME_it_ are observations for subject *i*_3_ at baseline and follow-up; *β*_0_ is the intercept; *β*_1_*, β*_2_*, β*_3_*, β*_4_*,* and *β*_5_*,* are the regression coefficients for *age, sex, pubertal stage, time, and study group x time*, respectively; *u*_*i*_ are random, subject specific intercepts and *v*_*i*_ are corresponding random slopes for follow-up time; and *ε*_*it*_ is the error for subject *i* at time *t*. Time was treated as a continuous variable to allow for a slight variation in follow-up time (1.8–2.5 years) among the children.

We used a Bayesian information criterion (BIC) as a measure of model adequacy. The BIC value penalizes the likelihood of the observed data based on the total number of parameters in a model. A lower BIC value indicates a better model with a better balance between complexity and good fit. We fitted all possible models with allowing or ignoring the possible clustering on subject and/or school level for each dependent variable. However, we a priori chose the model with the lowest BIC value as our final model for a given variable. Thus, we did not force the three-level data structure to our model since it did not improve model fit but resulted in unnecessary complexity.

The data for all lipids and lipoproteins showed the best fit with the model in which a random intercept and a random regression coefficient of time were modeled on the subject level using an independent variance structure, but no random effect for intercept or regression coefficient of time on the school level was included.

One of the typical problems related to the use of the time-by-study group interaction is the phenomenon of regression to the mean due to the differences between the intervention and control group at baseline. We had no differences in plasma lipids and lipoproteins between the study groups at baseline (*p* = 0.108–0.729). Therefore, we did not include the study group in the model to allow for the regression to the mean phenomenon. Instead, baseline values in the study groups are reflected in the intercept of the model.

In further analyses, we studied the possible confounding factors on the effects of the intervention on plasma lipids and lipoproteins adjusting the analyses for body fat percentage at both time points or for parental education level.

We also studied how physical activity, sedentary behavior, and diet contributed to the observed effects of the intervention using linear mixed-effect models adjusted for age at baseline, sex, and pubertal stage at both time points and entering the physical activity, sedentary behavior, and diet variables, according to the aims of the intervention, one by one as potential explanators for the effects. Changes in the regression coefficients after entering the physical activity, sedentary behavior, and diet variables in the models are presented to show the magnitude of the effect of these adjustments.

## Results

### Characteristics of children at baseline and 2-year follow-up

There were no differences in the characteristics of children between the intervention and control group at baseline (Table 1). Only 3% of children in the intervention group and 2% of children in the control group were pubertal at baseline; whereas, 23% of children in both groups had entered puberty by the 2-year follow-up.

**Table 1:** Characteristics of children at baseline

	Intervention group	Control group	*p* value
Number	306	198	
*Sex*			0.519
Girls	144 (47.1)	99 (50.0)	
Boys	162 (52.9)	99 (50.0)	
Age, y	7.6 (0.4)	7.6 (0.4)	0.499
*Parental education*^*1*^			
Vocational school or less	57 (18.8)	41 (20.7)	
Polytechnic	139 (45.9)	85 (42.9)	
University	107 (35.3)	72 (36.4)	0.783
Body height, cm	128.9 (5.5)	128.6 (5.9)	0.475
Body weight, kg	27.0 (4.8)	26.8 (5.3)	0.733
Body fat percentage, %^2^	19.7 (8.4)	20.0 (8.2)	0.771
BMI-SDS	− 0.2 (1.1)	− 0.2 (1.1)	0.679
Overweight or obese	41 (13.4)	25 (12.6)	0.802

Values are means (standard deviations) and* p* values from the* t* test for independent samples for continuous variables and number of children (%) and *p*-values from Chi-Square test for sex and being overweight or obese

Overweight or obesity was defined according to the International Task Force criteria (12)

*BMI-SDS*, body mass index standard deviation score based on Finnish reference values (11)

^1^*n* = 303 in intervention group, *n* = 198 in control group

^2^*n* = 296 in intervention group, *n* = 194 in control group

### Effects of intervention on concentrations of plasma lipids

Plasma LDL cholesterol concentration decreased in the intervention group but did not change in the control group ( − 0.05 mmol/L vs. 0.00 mmol/L, *p* = 0.040 for group*time interaction) adjusted for age at baseline, sex, and pubertal stage at both time points (Table 2). This effect remained statistically significant after further adjustments for body fat percentage (*p* = 0.028 for group*time interaction) and for parental education level (*p* = 0.036 for group*time interaction). The intervention had no effect on plasma concentrations of total cholesterol, HDL cholesterol, VLDL cholesterol, triglycerides, HDL triglycerides, or VLDL triglycerides after these adjustments (Table 2).

**Table 2:** Plasma lipids concentrations (mmol/L) at baseline and at 2-year follow-up and their changes (mmol/L) during 2-year follow-up

	Intervention group			Control group			
	Baseline	2-year follow-up	2-year change	Baseline	2-year follow-up	2-year change	*p* for group x time interaction
Plasma total cholesterol	4.26 (4.19, 4.34)	4.32 (4.24, 4.40)	+ 0.06	4.29 (4.21, 4.37)	4.32 (4.22, 4.42)	+ 0.03	0.629
Plasma LDL cholesterol	2.35 (2.29, 2.41)	2.30 (2.23, 2.37)	− 0.05	2.37 (2.30, 2.44)	2.37 (2.29, 2.46)	0.00	0.040
Plasma HDL cholesterol	1.60 (1.57, 1.64)	1.64 (1.60, 1.69)	+ 0.04	1.59 (1.55, 1.63)	1.62 (1.57, 1.67)	+ 0.03	0.810
Plasma VLDL cholesterol	0.12 (0.11, 0.13)	0.13 (0.12, 0.14)	+ 0.01	0.13 (0.12, 0.15)	0.14 (0.13, 0.16)	+ 0.01	0.427
Plasma triglycerides	0.59 (0.56, 0.61)	0.62 (0.58, 0.66)	+ 0.03	0.62 (0.59, 0.66)	0.64 (0.60, 0.68)	+ 0.02	0.898
Plasma HDL triglycerides	0.15 (0.15, 0.16)	0.15 (0.15, 0.16)	0.00	0.16 (0.15, 0.16)	0.16 (0.15, 0.17)	0.00	0.323
Plasma VLDL triglycerides	0.27 (0.25, 0.30)	0.29 (0.26, 0.32)	+ 0.02	0.30 (0.26, 0.33)	0.30 (0.27, 0.33)	0.00	0.849

Values are unadjusted means (95% confidence intervals) at baseline and at 2-year follow-up as well as 2-year changes in the means. P-values for the group x time interaction are from linear mixed-effect models adjusted for age at baseline, sex, and pubertal stage at both time points. Intercept and time were included as random effects into the models on the subject level

*LDL* low-density lipoprotein, *HDL* high-density lipoprotein, *VLDL* very-low-density lipoprotein

### Factors contributing to the effects of intervention on plasma LDL cholesterol concentration

The consumption of high-fat (60–80%) vegetable oil-based spreads explained the most of the effects of the intervention on plasma LDL cholesterol concentration (+ 47% change in regression coefficient) followed by moderate-to-vigorous physical activity (+ 30% change), sedentary time (+ 30% change), light physical activity (+ 29% change), and the consumption of butter-based spreads (+ 24% change), low-fat (< 1%) milk (+ 19% change), and high-fat (≥ 1%) milk (+ 16% change) adjusted for age at baseline, sex, and pubertal stage at both time points (Table 3). Other dietary factors explained less of the effects of the intervention on plasma LDL cholesterol concentration (Table 3).

**Table 3:** Regression coefficients for intervention effect on plasma LDL cholesterol concentration before and after entering physical activity, sedentary behavior, and diet variables according to the aims of the intervention one by one as potential factors contributing to the effects

	β	Change in β	*p* for group x time interaction
Model without lifestyle adjustments	− 0.0385		0.040
*Adjustment for physical activity*			
Moderate-to-vigorous physical activity, min/d	− 0.0268	+ 30%	0.186
Light physical activity, min/d	− 0.0274	+ 29%	0.179
Sedentary time, min/d	− 0.0270	+ 30%	0.187
*Adjustment for food consumption*			
Butter-based spreads, g/day	− 0.0294	+ 24%	0.158
High-fat (≥ 1%) milk, g/day	− 0.0325	+ 16%	0.122
Low-fat (< 1%) milk, g/day	− 0.0312	+ 19%	0.137
Red meat, g/day	− 0.0356	+ 8%	0.088
High-fat (60–80%) vegetable oil-based spreads, g/day	− 0.0203	+ 47%	0.335
Vegetable oils, g/day	− 0.0356	+ 8%	0.089
Fish, g/day	− 0.0361	+ 6%	0.085
Vegetables, fruit and berries, g/day	− 0.0379	+ 2%	0.072
Low-fiber grain products, g/day	− 0.0348	+ 10%	0.097
High-fiber grain products, g/day	− 0.0354	+ 8%	0.090
Sugary products^a^, g/day	− 0.0358	+ 7%	0.087

Values are regression coefficients (β) from the linear mixed-effects model adjusted for age at baseline, sex, and pubertal stage at both time points, and additionally for each food consumption and physical activity variable separately. Intercept and time were included as random effects into the models on the subject level

^a^Including sugar-sweetened beverages, candies, chocolate, ice cream and puddings

## Discussion

This 2-year controlled lifestyle intervention study demonstrated that the individualized and family-based physical activity and dietary intervention resulted a small decrease in fasting plasma LDL cholesterol concentration in a population sample of mainly prepubertal children. We have previously reported that our intervention increased self-reported physical activity and the consumption of vegetables, high-fat vegetable oil-based spreads and low-fat milk and decreased the consumption of butter-based spreads [13]. In the present study, we observed that the consumption of high-fat vegetable oil-based spreads was the strongest contributor for the effect of intervention on plasma LDL cholesterol. The changes in the consumption of high-fat milk, low-fat milk, and butter-based spreads as well as in physical activity also partly explained the intervention effect.

Some previous physical activity or dietary interventions have been observed to be beneficial for the reduction of plasma lipids and lipoproteins in children with obesity [5] or hypercholesterolemia [6]; whereas, other lifestyle interventions have shown little or no effects on plasma lipid concentrations [5, 7, 8]. Moreover, lifestyle interventions aiming at preventing hypercholesterolemia or dyslipidemia in general populations of children are scarce. In one study, an individualized and family-based dietary intervention that was initiated in infancy and included biannual low saturated fat dietary counseling attenuated the increase in serum LDL cholesterol concentration in a general population of children, most of whom had not yet entered puberty [9].We found a small decrease of 0.05 mmol/l in plasma LDL cholesterol concentration in the physical activity and dietary intervention group and no change in the control group during the 2-year follow-up in a population sample of children with relatively low concentrations of LDL cholesterol and low prevalence of overweight at baseline. However, not even an intervention based on a cholesterol-lowering diet among children with hypercholesterolemia has shown a much stronger effect on plasma LDL cholesterol concentration than the effect of our combined physical activity and dietary intervention in a population sample of children [6]. Even slightly increased serum LDL cholesterol concentration in childhood has been associated with increased carotid intima–media thickness, an indicator of preclinical atherosclerotic cardiovascular disease, in adulthood [2, 3]. Therefore, the beneficial effect of our intervention on plasma LDL cholesterol concentration could decrease the future risk of atherosclerotic cardiovascular disease development, particularly if continued until adulthood. However, the long-term clinical significance of such a small change in plasma LDL cholesterol concentration remains unknown.

Improvement in the quality of dietary fat is known to decrease plasma LDL cholesterol concentration by plausible biological mechanisms. While a lower intake of saturated fat decreases the formation of LDL particles and increases its turnover, a higher intake of unsaturated fat increases the number of hepatic LDL cholesterol receptors and enhances the clearance of LDL particles [27]. We have previously reported that high-fat milk and butter-based spreads were among major food sources of saturated fat and that high-fat vegetable oil-based spreads were among major sources of polyunsaturated fat in children [28]. Therefore, we suggest that replacing high-saturated fat products by low-fat milk and butter-based spreads by high-fat vegetable oil-based spreads in the diet can be used to decrease plasma LDL cholesterol concentration in children. A higher intake of soluble fiber has also been shown to reduce plasma LDL cholesterol concentration in adults [29] and in children [30]. Nevertheless, we found that the changes in fiber-rich foods, such as high-fiber grain products and vegetables, fruit, and berries, explained the effect of the intervention on plasma LDL cholesterol concentration only modestly.

The biological mechanisms by which physical activity could decrease plasma LDL cholesterol concentration are less clear than those of the quality of diet. Such mechanisms involve changes in the activities of key enzymes in lipid metabolism, including lipoprotein lipase and hepatic lipase, as well as LDL receptors [31]. We found that not only the change in the quantity and quality of fat but also the change in physical activity, regardless of its intensity, explained the effect of the intervention on plasma LDL cholesterol concentration.

Lifestyle interventions should not only be effective but also applicable in everyday conditions, including healthcare, and should be acceptable to children and their parents to maintain long-term adherence. We designed the physical activity and dietary intervention to be applicable in everyday conditions. For example, we organized exercise clubs at schools directly after school hours to make it easier for the children to attend. Moreover, we emphasized the individual needs of the families and the parental involvement to improve adherence to lifestyle intervention [32]. Only 15% of the children in the intervention group dropped out during the 2-year follow-up, and almost 90% of the children and their parents participated in all six physical activity and dietary counseling sessions. These findings suggest that the lifestyle intervention was well accepted and could be applicable in everyday conditions, including healthcare.

A strength of our study is that we had the opportunity to investigate the effects of a long-term physical activity and dietary intervention on plasma lipid concentrations in a general population of mainly prepubertal children having a prevalence of overweight similar to the national reference population [14]. Moreover, though the proportion of pubertal children was similar in the intervention and control group at baseline and 2-year follow-up, we controlled for pubertal stage because it could have obscured some of the health effects of lifestyle intervention, for example by decreasing plasma LDL cholesterol concentration [33]. Another strength of the study is that we objectively measured physical activity and sedentary time using a combined movement and heart rate sensor. Using objective measurements diminishes the recall bias and coding errors. A weakness of the study is that, although carefully guided and checked by clinical nutritionists, diet was assessed using food records reported by the parents, which may have caused misreporting. Another weakness is that we did not randomly allocate the participants in the intervention and control group, and therefore, causality cannot be established. Instead, we divided the children into the groups according to schools. However, this allowed us to organize after-school exercise clubs carried out at schools only for the intervention group and to avoid non-intentional intervention in the control group. We also matched the children in the intervention and control group according to the size and location of the schools to minimize differences in baseline characteristics between the groups. There were no differences in baseline characteristics between the intervention and control group, suggesting that we succeeded in avoiding selection bias. We also tested the possible clustering effect of the schools in the linear mixed-effects model analyses but did not include school as a random factor in the final models because of negligible clustering effect. Diet and physical activity on the day before blood sampling may have affected plasma lipid concentrations and confounded the statistical analyses and thus underestimated the true long-term effects of the intervention. The observed intervention effect may be dependent on the existing lifestyle factors, such as food consumption, and may not be generalizable to children in other countries. Moreover, the observed change in LDL cholesterol concentration was small and, therefore, may be due to confounding or bias.

Our study revealed that the combined physical activity and dietary intervention resulted a small decrease in plasma LDL cholesterol concentration during 2 years in a general population of mainly prepubertal children. If maintained in the long term, the beneficial effects of the physical activity and dietary intervention on plasma LDL cholesterol concentration may be meaningful for the prevention of atherosclerotic cardiovascular diseases in adulthood. Future lifestyle interventions aiming at reducing plasma LDL cholesterol concentration in general populations of children should focus on improving the quality of dietary fat in milk and spreads and increasing physical activity regardless of its intensity.

## Abbreviations

BMI-SDS: Body mass index standard deviation score
BIC: Bayesian information criterion
MET: Metabolic equivalent of task
PANIC: Physical activity and nutrition in children
